# Longitudinal assessment of daily activity patterns on weight change after involuntary job loss: the ADAPT study protocol

**DOI:** 10.1186/s12889-017-4818-2

**Published:** 2017-10-10

**Authors:** Patricia L. Haynes, Graciela E. Silva, George W. Howe, Cynthia A. Thomson, Emily A. Butler, Stuart F. Quan, Duane Sherrill, Molly Scanlon, Darlynn M. Rojo-Wissar, Devan N. Gengler, David A. Glickenstein

**Affiliations:** 1 Health Promotion Sciences, Mel and Enid Zuckerman College of Public Health, University of Arizona, 1295 N. Martin Ave, Tucson, AZ 85724 USA; 2 College of Nursing, University of Arizona, 1305 N. Martin Ave, Tucson, AZ 85721 USA; 3 Department of Psychology, Columbian College of Arts and Sciences, George Washington University, 2125 G St NW, Washington, DC 20052 USA; 4 Family Studies & Human Development, University of Arizona, 650 N. Park Ave, Tucson, AZ 85721 USA; 5 College of Medicine, University of Arizona, 1501 N. Campbell Ave, Tucson, AZ 85724 USA; 6 Division of Sleep and Circadian Disorders, Brigham and Women’s Hospital, Harvard Medical School, 164 Longwood Ave., Boston, MA 02115 USA; 7 Biostatistics, Mel and Enid Zuckerman College of Public Health, University of Arizona, 1295 N. Martin Ave, Tucson, AZ 85724 USA; 8 Department of Mathematics, University of Arizona, 617 N. Santa Rita, Tucson, AZ 85721 USA

**Keywords:** Obesity, Sleep, Stress, Unemployment, Social rhythms

## Abstract

**Background:**

The World Health Organization has identified obesity as one of the most visible and neglected public health problems worldwide. Meta-analytic studies suggest that insufficient sleep increases the risk of developing obesity and related serious medical conditions. Unfortunately, the nationwide average sleep duration has steadily declined over the last two decades with 25% of U.S. adults reporting insufficient sleep. Stress is also an important indirect factor in obesity, and chronic stress and laboratory-induced stress negatively impact sleep. Despite what we know from basic sciences about (a) stress and sleep and (b) sleep and obesity, we know very little about how these factors actually manifest in a natural environment. The Assessing Daily Activity Patterns Through Occupational Transitions (ADAPT) study tests whether sleep disruption plays a key role in the development of obesity for individuals exposed to involuntary job loss, a life event that is often stressful and disrupting to an individual’s daily routine.

**Methods:**

This is an 18-month closed, cohort research design examining social rhythms, sleep, dietary intake, energy expenditure, waist circumference, and weight gain over 18 months in individuals who have sustained involuntary job loss. Approximately 332 participants who lost their job within the last 3 months are recruited from flyers within the Arizona Department of Economic Security (AZDES) Unemployment Insurance Administration application packets and other related postings. Multivariate growth curve modeling will be used to investigate the temporal precedence of changes in social rhythms, sleep, and weight gain.

**Discussion:**

It is hypothesized that: (1) unemployed individuals with less consistent social rhythms and worse sleep will have steeper weight gain trajectories over 18 months than unemployed individuals with stable social rhythms and better sleep; (2) disrupted sleep will mediate the relationship between social rhythm disruption and weight gain; and (3) reemployment will be associated with a reversal in the negative trajectories outlined above. Positive findings will provide support for the development of obesity prevention campaigns targeting sleep and social rhythms in an accessible subgroup of vulnerable individuals.

## Background

Excess body weight is a major public health crisis in the United States and worldwide [1, 2]. Both modifiable and unmodifiable factors influence obesity risk, making obesity a complex and multifaceted diagnosis. While much work has examined singular causes, fewer studies have examined the *interactions* of social, psychological, and biological variables. The purpose of the Assessing Daily Activity Patterns through occupational Transitions (ADAPT) study is to examine weight change as a function of the changing interrelationships between daily behavioral patterns and sleep in the aftermath of a stressful life event – involuntary job loss.

In May 2017, approximately 6.9 million individuals in the U.S. were unemployed with 3.3 million of these individuals classified as having lost their jobs [3]. Despite a recent improvement in U.S. unemployment rates, job loss remains a stressful life event that is associated with weight gain [4–6] and related negative health outcomes [7–12]. Previous research has shown job loss signals a cascade of negative mental health outcomes, often associated with economic resource depletion [13]. In addition, job loss impacts a person’s daily activities, sleep patterns, and social rhythms. Social rhythms are daily habitual behaviors, such as mealtimes, physical activities, and sleep/wake times, occurring in rhythmic 24 h cycles [14]. Social rhythms are tied to the 24 h light/dark cycle. As such, they alter our retinal exposure to light (e.g., via photoperiod length and light exposure intensity) or the circadian system response to light (e.g., moderating sensitivity to light) [15], thereby impacting the expression of circadian rhythms like sleep and motor activity. Regular and active social rhythms are associated with good quality sleep [16, 17]. To our knowledge, no research has prospectively examined sleep or social rhythms after job loss, although changes in work hours are associated with disrupted social rhythms [18]. Epidemiological studies in the U.S. [19, 20], Australia [21] Korea [22], China [23], Japan [24] and Finland [25] have found that unemployment is associated with sleep complaints. Unemployed individuals are also more likely to have short or long sleep durations [22] and insomnia diagnoses characterized by difficulties maintaining sleep [23, 24].

Insomnia and short sleep duration are also associated with obesity [26–28] and obesity-related health outcomes, such as hypertension [29], diabetes [30, 31], and mortality [32]. Yet, the relationship between sleep and obesity has been inconsistent, potentially due to methodological issues [33, 34]. Most studies have examined the relationship between sleep and weight gain using cross-sectional or retrospective cohort designs. The majority of studies employing a prospective design [35–40] assessed sleep duration via global interview questions, which have significant drawbacks [41]. Three studies obtained objective measures of sleep duration and body mass index at baseline and approximately 5 [42, 43] to 7.5 years [44] later. In two studies, findings were positive for a cross-sectional relationship between sleep duration and body mass index (BMI) but negative for a prospective association [42, 43]. Similarly, Vgontzas and colleagues [44] found no association between sleep duration, assessed by one night in-lab polysomnography, and later obesity. Self-reported sleep disturbance and self-reported lower sleep duration did predict later obesity -- but only when emotional distress was excluded from the model. Results from this study suggest that emotional distress may mediate the relationship between poor subjective sleep and later obesity.

While these studies represent a major advance forward, they were limited by an in-lab sleep assessment largely susceptible to atypical sleep [44], an assessment of BMI only [42–44] and the use of one distant follow-up time point [42–44], which does not allow for the examination of weight gain trajectories. The ADAPT project employs a multiple assessment longitudinal design that allows the use of growth curve analytic techniques for examining sleep and weight gain trajectories over time. Furthermore, the longitudinal design will allow an investigation of the temporal sequencing of sleep and weight gain, which will strengthen the ability to make causal inferences.

In this paper, we present the protocol for ADAPT, an ongoing closed cohort study. It is anticipated that the results from this study will advance obesity research by translating laboratory findings in sleep and weight gain to the natural environment. Moreover, results from this study will inform the development and testing of obesity prevention campaigns targeting sleep and social rhythms in a substantial number of accessible and vulnerable individuals.

## Methods

### Aims

**Fig. 1:**
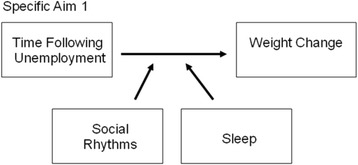
Conceptual model for ADAPT Specific Aim 1

**Fig. 2:**
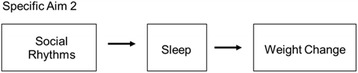
Conceptual model for ADAPT Specific Aim 2

The overall objective of the ADAPT study is to explore whether social rhythms and sleep operate as mechanisms of weight gain following involuntary job loss. The specific aims of ADAPT are to examine whether:

- Specific Aim 1. Initial disruptions in social rhythms and sleep moderate weight change trajectories following job loss. See Fig. 1.
- Specific Aim 2. Short sleep and sleep disturbances mediate the relationship between disrupted social rhythms and weight change following unemployment. See Fig. 2.
- Specific Aim 3. Reemployment is associated with a stabilization of social rhythms, sleep, and weight change.

Models proposed in Specific Aims 1 and 2 will control for reemployment. Also, we will be employing gold standard measures of stress and psychological distress that will allow us to control for the negative economic and emotional consequences of job loss. Last, caloric intake and energy expenditure are assessed for secondary analyses. These secondary analyses will be conducted in order to translate weight gain findings to core behaviors of energy balance, providing valuable data to inform future behavioral interventions.

### Design

Participants are evaluated on social rhythms, sleep, and weight at 6 time points over the course of 18-months. This longitudinal design was chosen in order to (a) model the accelerating changes of the three variables over time including any potential curvilinear relationships (such as those proposed between sleep duration and weight gain [27]), (b) provide sufficiently frequent assessments to correctly identify the temporal sequence of improvement associated with reemployment, and (c) balance participant burden. Each time point requires a two-week period to provide an acceptable time frame for assessments (e.g., USDA Multi-pass dietary recall [45] Social Rhythm Metric (SRM) [46]). The study design was developed and is reported according to the Strengthening the Reporting of Observational Studies in Epidemiology (STROBE) statement [47].

### Recruitment

The primary approach to recruitment for ADAPT is through the Arizona Department of Economic Security (AZDES). On a weekly basis, recruitment letters describing the study are included in packets of information sent out to all individuals applying for Unemployment Insurance (UI) within Tucson and neighboring metropolitan areas (approximately 380 per week). This contractual agreement required significant time and attention to assure the protection of private information. Therefore, we also posted recruitment fliers at the local libraries and on Craigslist, a popular U.S. website for individuals searching for employment, in order to expedite the start of recruitment. Recruitment began in October 2015 with the goal of recruiting 322 participants. We anticipated a screen fail rate of 22% in order to achieve a final, anticipated sample of 250 participants who complete the baseline assessment.

### Eligibility criteria

All participants are between the ages of 25-60 years, as age above or below this range may have substantial impact on sleep [48, 49] and social rhythms [50]. To complete forms and participate in interviews, individuals are required to fluently speak, read, and write English. To detect a change in social rhythms associated with job loss, individuals must have been working at their previous job for at least 6 months for 30 or more hours per week and have been laid off or terminated from their place of employment within the last 90 days. Job-related eligibility criteria were informed by previous research [51] and the proposed model, which suggest that 90 days is a conservative window of time to detect sleep-related consequences of job loss.

Participants are excluded for engaging in any treatments likely to impact weight gain or energy balance-related behaviors (e.g., weight loss surgery, recent participation in weight loss program, recent use of weight loss medications). In addition, participants are excluded for a number of variables likely to negatively impact sleep as detailed in Table 1. To ensure sufficient generalizability to the population of individuals who have involuntarily lost their jobs, the study allows participation by individuals taking medications for sleep, including medical marijuana, and individuals with a history of shift work. Individuals are not enrolled if they have engaged in shift work within the past 30 days, if they meet criteria for substance abuse or dependence, or if they have taken narcotics for sleep and pain within the last 6 weeks. Random urine drug screening is performed with the suspicion of drug use.

**Table 1:** Exclusion Criteria

Construct	Criteria
Previous job	Quit Position
	Layoff is temporary
	Seasonal employment
	Overnight shift work, last 30 days
	Previous plan to retire within next 2 years
Time since job loss	Accepted offers of new job
	Part-time work >5 h per week
Weight	Weight loss medication
	Weight loss surgery
	Participation in weight loss program
	Eating disorder
Sleep	Pregnancy or ≤3 months postpartum
	Medical sleep disorder
	Major medical disorder
	Severe mental illness
	Current substance or alcohol use disorder, last 3 months
	Moderate to severe sleep apnea defined as an Apnea Hypopnea Index (AHI) ≥ 15

Participants are excluded if they are homeless because of difficulty in maintaining contact for follow-up assessments. They are also excluded if they report a prior felony charge or if they demonstrate a potential threat to study staff or equipment, as assessed by a licensed clinical psychologist.

### Sample size calculation

To achieve 80% power to detect a difference in BMI of 1.0, we must enroll a total of *N* = 231 individuals eligible for study participation. We will over-enroll to 250 eligible individuals to account for attrition, which provides power of 83% to detect significant weight gain. Sample size estimates were made using pilot data of BMI that were collected prospectively on 20 adult participants over a 12-month period providing 40 observations total (PI: Haynes). Employment status, psychological distress, and social rhythm measurements were obtained at baseline. Total sleep time (TST) and wake after sleep onset (WASO) were obtained at baseline and follow-up. These pilot data were used to estimate the expected mean difference (Δ), between subject variance (σ^2^), and intra class correlation (ρ) between repeated observations, values necessary for sample size estimation. Sample size estimates were made assuming normally distributed data using the following equation, (*N* = 2(Z _1-α/2_ + Z_β_)^2^ σ^2^(1 + (m-1)ρ)/mΔ^2^). Models were adjusted for WASO, psychological distress, and social rhythm, where m = number repeated observation = 5, σ^2^ between subject variance = 29.70, ρ = correlation between observations = 0.987, α = 0.05, and Δ = mean difference = 1.0. We calculated power separately for TST and WASO so that we may take the most conservative estimate. Power analysis estimates will be repeated at the conclusion of data collection.

### Procedure

Individuals meeting inclusion criteria via a phone screen are scheduled for an in-person screening visit (Visit 0, V0). At this visit, study staff inform participants about the study’s purpose and obtain written informed consent to participate. To meet inclusion criteria, study staff administer a battery of valid and reliable measures, including: demographics, the Charlson Comorbidity Index [52], a medical history form, the Duke Sleep Interview (DSI) for Sleep Disorders [53], the Mini International Neuropsychiatric Interview (MINI) [54], and a past employment interview. Participants are given instructions on the use of the ApneaLink Plus™ device (ResMed), which they wear while sleeping in their home that night. Study staff retrieve the ApneaLink Plus™ device the following morning for immediate scoring by a registered polysomnographic technologist and interpretation by a physician board-certified in sleep medicine (SFQ). During the V0 interview, participants are also given the Arizona Activity Frequency Questionnaire (AAFQ) [55], a Job Loss Stressors Survey, and the Morningness Eveningness Questionnaire [56] to complete and bring back to their next scheduled interview (Visit 1, V1).

Eligible participants then continue with an initial baseline assessment (V1), where measurements of adiposity and body composition are completed (see Weight Gain measures below). Participants also receive a venous fasting blood draw, and provide an optional hair sample for subsequent biomarker analysis. Participants are then offered the opportunity to eat breakfast. They are given instructions on the remote food photography method (RFPM) application, and they practice using this application before and after their breakfast meal. After breakfast, participants are administered the life events interview and complete a series of questionnaires about sleep, diet, physical activity, psychological distress and locus of control, adverse childhood experiences, and food security (see Measures below). At the end of the visit, participants are instructed to wear (1) an actigraph (Actiwatch®) over their sleeves on their non-dominant wrist and (2) a different actigraph (Actical®) on a waistband on their right hip. Participants are given a smart tablet with instructions on the completion of the Daily Sleep Diary each morning and the SRM each evening via a website application. Participants are informed on the procedure for the 3-day USDA multi-pass dietary recall and the 3-day physical activity recall. Recalls are scheduled for 3 days over the next 2 weeks (dates and times), and participants are instructed take pictures of meals, beverages, and snacks before and after consumption to facilitate their memory and intake assessment.

Over the next 2 weeks, participants wear the devices and complete the daily diaries every morning and evening. Study staff monitors completion of the diaries on a daily basis through a website portal and call participants if diaries are not completed on time, thereby reducing recall bias and missing data. Participants return the devices at the end of the two-week period and are scheduled for an additional 5 visits (V2 – V6) occurring 3 - 4 months apart, where the same battery of assessments is administered. In addition, participants are administered additional interviews assessing for changes in employment and financial status, demographics, and medical status. Brief versions of the DSI and MINI are administered to assess for changes in sleep and mental disorders.

Participants are provided with cash or gift cards to compensate for their time participating in each visit activity. Participation in all visits and volunteering hair collection provides a maximum compensation of $1020, along with 7 breakfast meals. A summary of their sleep report and referral is provided in the event of a positive screening for sleep disordered breathing.

### Measures

#### Weight gain

Height (cm) and weight (kg) are measured at V1 using standardized study protocols and with subsequent measures of weight taken at each of the following time points (V2 – V6). Total body fat, percent body fat, lean mass, percent water, and BMI from Bioelectrical Impedance Analysis (BIA) are also calculated. BIA provides an objective measure of body fat that is easy to conduct and does not involve radiation exposure associated with dual-energy X-ray absorptiometry. Waist circumference (cm) is also obtained as the primary measure of central adiposity using standardized protocols. In addition, we plan to compute clinically relevant indicators of obesity for follow-up analyses (e.g., ≥ 5% increase body weight, movement into higher BMI categories, and waist circumference clinical cutoffs).

#### Social rhythms

The SRM [46] is a valid and reliable [57] self-report instrument designed to measure daily habitual behaviors and interactions. The main index derived from this instrument represents the *regularity* of an individual’s life. However, the *volume of activities* performed per week will also be computed and separately examined.

#### Sleep

Consistent with insomnia research consensus panel recommendations [58], we are assessing disturbances in sleep via the research consensus Daily Sleep Diary (DSD) [58] and actigraphy [59, 60]; sleep quality via the Pittsburgh Sleep Quality Index (PSQI) [61]; and sleep timing preferences via the Morningness Eveningness Questionnaire (MEQ) [56]. The primary measure of subjective sleep is the DSD, a valid and reliable assessment of sleep onset latency (SOL), TST, total time in bed (TIB), sleep efficiency (SE), WASO, and sleep quality [62]. The PSQI and MEQ will be used in secondary analyses; they are valid and reliable self-report questionnaires assessing sleep quality and sleep timing [56, 61]. In addition, trained research staff administer an abbreviated version of the DSI [53], a semi-structured interview, to assess insomnia diagnosis using International Classification of Sleep Disorders (ICSD) criteria.

The Actiwatch Spectrum Plus® (Phillips Respironics) is the primary measure of objective sleep. Participants are instructed to wear the Actiwatch® with a light monitor on their nondominant wrist for 2 weeks [60] in order to obtain an objective measure of sleep. To facilitate scoring, participants are asked to press an event marker button to indicate the time they retire to bed with the intention to sleep and the time they arise from bed at the end of their sleep period. Activity plots are being examined to determine SOL, TIB, TST, WASO, SE and circadian rhythm variables. In addition to sleep, overall exposure to ambient light is gathered for the out-of-bed and in-bed periods, which will be used for secondary analyses. Actigraphy provides valid and reliable measures of sleep-wake organization with high rates of agreement (above 80%) with polysomnography [59, 60]. Total sleep time and wake time after sleep onset are the main sleep outcomes. All other indices, including sleep quality from the DSD and PSQI, are being used for secondary analyses. In addition, Actiwatch® indices include cosinor analysis variables and ambient light exposure to provide data for follow-up and exploratory analyses.

#### Dietary intake and physical activity

Behavioral and dietary specialists conduct assessments of dietary intake and physical activity using a series of three randomly scheduled 24-h recall interviews [45]. Data are collected using the gold-standard USDA Multi-pass Dietary recall methodology [45] and during the call simultaneously enter data into the Nutrient Database System of the University of Minnesota for nutrient analysis. These interviews provide variables indicating nutrient quantity and quality of food eaten, substances consumed (including alcohol, nicotine, and caffeine), the timing of consumption, and energy expenditure from physical activity in metabolic equivalents (METs). To facilitate the recall, participants capture images of food and beverage before and after consumption via the Remote Food Photography Method (RFPM) [63, 64].

The primary objective measure of energy expenditure is daily physical activity data gathered from the Actical® (Phillips Respironics), an accelerometer participants wear over the right hip on an elastic belt [65]. To facilitate scoring, participants are instructed to use the event marker before and after they engage in physical activity. Accelerometer output predicts daily energy expenditure and time spent in four physical activity intensity ranges, specified in METs: sedentary (1–1.5 METs), light (1.5–3 METs), moderate (3–6 METs), and intense/vigorous (>6 METs) [66]. The Actical® has good intra- and inter-instrument reliability [67] and good overall agreement with total energy expenditure measured using a whole-room indirect calorimeter, especially in time spent in moderate to intense physical activity [68]. The International Physical Activity Questionnaire [69] and the Arizona Activity Frequency Questionnaire (AAFQ) [55] are administered as secondary measures of physical activity.

#### Reemployment

The Reemployment Status interview is a demographic interview administered at follow-up assessments to assess changes in work status by date. The main index of reemployment is a dichotomous variable indicating whether the person is employed ≥30 h per week.

#### Eligibility criteria, covariates

##### Job loss stressors

This interview examines the nature and characteristics of prior work, including information about prior income, part-time/full-time status, temporary/permanent status, type of employment, benefits, as well as perceived work stress [70].

##### Demographics, medical history

At the screening assessment, participants receive a demographic interview and the Charlson Comorbidity Index (CCI) [52], a self-report questionnaire describing severity of 19 health conditions. The CCI is used to guide a medical history interview about lifetime and current medical conditions, treatment history, and use of medications, among others.

##### Life events interview

Consistent with previous work [71, 72], trained study staff administer a semi-structured life events interview using the Psychiatric Epidemiology Research Interview Life Events Scale [73] to gather information about the nature and date of life events, the length of the event, and the circumstances in which the event occurred (e.g., if the participant is the sole wage earner in the family, consequences of job loss on housing, finances, daily routine). Life events interviewing is done to acquire information to control for the presence of other stressful life events including (a) the contextual impact of job loss, financial strain, and other events or difficulties, and (b) the severity of social rhythm disruption specifically attributable to job loss and other events. A trained consensus panel rates the threat level of each life event and long-term difficulty. Raters employ a standardized set of criteria set forth in the Life Events and Difficulties Schedule (LEDS) rating system [74] to assess the event’s level of impact on a typical person under similar circumstances.

The adjunct Social Rhythm Disruption (SRD) rating criteria [75] assess how likely the event is to directly cause a substantial change in routine leading to sleep disruption or a change in the sleep/wake routine. All raters are trained to an adequate level of agreement (kappa > 0.8). The LEDS interviewer-based life events assessment approach is considered the gold standard in life events assessment [76], because it does not introduce systematic bias associated with stress perception. Stress perception is an important consideration in the assessment of stress severity, since distress often leads to over-reporting of negative items [77].

##### Sleep disorders

Participants receive the ApneaLink Plus™ (ResMed), an ambulatory screening device for sleep disordered breathing that is administered in the participant’s home. It records respiratory effort and heart rate via an inductance chest belt, oxygen saturation by pulse oximetry, and air flow by nasal cannula connected to a pressure transducer to generate an Apnea-Hypopnea Index (AHI). The Apnealink Plus™ has a high level of sensitivity (>80%) at all AHI levels [78], and a positive predictive value of greater than 80% at AHI levels of 10 or more [79]. For other sleep disorders, trained research staff administer the DSI [53], a semi-structured interview allowing a systematic review of medical sleep disorder symptoms using the International Classification of Sleep Disorders (ICSD)-2 criteria [53] (with experimental questions for ICSD-3 criteria, since this assessment had not been updated at the time of study implementation).

##### Psychiatric diagnosis and psychological distress

Because few studies examining sleep and weight gain have assessed psychological distress, it was premature to theorize about the temporal sequencing of how this variable interacts with social rhythms, sleep, and weight gain over time. Instead, we made the prudent decision to control for psychological distress with the hope that inclusion of this construct allows for follow-up analyses to inform future theoretical models. To assess for these constructs, we administer the valid and reliable MINI [54, 80, 81] to exclude individuals with severe mental illness (schizophrenia, bipolar disorder, alcohol and substance use disorders) and control for the development of other psychiatric disorders at each time point. To separately control for psychological distress, we will create a composite variable to control for depression symptoms (Beck Depression Inventory (BDI-II) [82]), anxiety symptoms (Beck Anxiety Inventory (BAI) [83]), and perceived stress (Perceived Stress Scale (PSS) [84]).

#### Exploratory variables

##### Surveys

The following three scales are administered to inform future work on unemployment, stress, and obesity, including: (1) Rotter’s 10-item Locus of Control Scale [85] to assess the degree to which a person views environmental events as being under their personal control; (2) Adverse Childhood Experiences (ACE) Survey to assess exposure to potentially traumatic events in childhood [86]; and (3) the US Household Food Security Survey Module [87] to assess access to sufficient affordable and nutritious food. Variables computed from each of these surveys have been previously associated with poor health outcomes, although few studies have examined these variables in the context of involuntary unemployment and sleep specifically.

##### Biomarker collection

A 12-h fasting blood draw is performed the morning of each assessment using the Vacutainer system and following universal precautions to gather data for future analysis of biomarkers implicated in appetite regulation, satiety, stress, inflammation, and the metabolic syndrome. In addition, participants are offered the opportunity to provide a ½ - 1 cm in diameter hair samples on selected visits. Biomarker analysis for future projects will be informed by theory.

### Data analysis plan

Preliminary analyses will be conducted to combine variables into composites where appropriate and to ensure that distributional assumptions, scale reliability, and a Missing at Random (MAR) pattern for missing data are met. If MAR assumptions are violated, we will consider using methods such as pattern mixture modeling to explore the impact of non-ignorable missingness. We will also explore whether potential covariates and predictors (see above, *Covariates*) are related to the central outcome variables.

For Aim 1, we will test a sequence of increasingly complex growth curve models, beginning with a model specifying that the weight composite does not change reliably over time. Subsequent models will systematically refine this model to establish the functional form of weight change, including predictors and covariates. As part of this process we will assess whether the association between sleep and weight gain, or social rhythms and weight gain, are better described by a linear or curvilinear relationship (e.g., both short and long sleep, or regular or irregular rhythms, predicts weight changes [88]). Nested alternative models will be compared using likelihood-ratio tests [89], with a focus on the interactions of initial levels of social rhythms and sleep with trajectories over time for weight gain (for Aim 1). For Aims 2 and 3, our approach will involve multivariate extension of multilevel models (e.g., random effects models) where time is nested in variables which is nested in persons (Bauer, Preacher, and Gil [90]), which provides a robust method for evaluating the patterns of lower-level mediation. In addition, we plan to test plausible alternative models to provide valuable information about the temporal sequencing of proposed variables. Models will be compared using standard fit statistics for non-nested models (e.g. root mean square error of approximation, Akaike information criterion, Bayesian information criterion, and others). For Aims 1 and 2, reemployment and psychological distress will be included as a time-varying predictors. For secondary aims, we will repeat all analyses separately substituting the weight composite variable with dietary intake and energy expenditure variables.

## Discussion

While much has been accomplished examining economic resource depletion and negative changes in personal status and identity after job loss, less research has examined changes in daily activity and structure. The ADAPT study aims to contribute to the scientific literature by examining how the proposed loss of daily activity and time structure resulting from job loss impacts sleep and weight change over time. Importantly, the longitudinal data collected will support a rigorous approach to the development of future interventions targeting weight, daily rhythms, and sleep in this at-risk group.

A major innovation of the ADAPT study is the employment of state-of-the-art assessments for weight gain, dietary intake, and energy expenditure. There is a well-documented bias for individuals to under-report weight and over-report height, seriously compromising the ability to determine objective weight gain in unemployed individuals from epidemiological samples [91]. Moreover, central obesity is a stronger risk factor than BMI for cardiovascular disease, type 2 diabetes mellitus [92], and mortality [93]. The ADAPT study employs measures of abdominal adiposity and the gold standard 24-h dietary and physical activity recall interviews, which will fill a unique gap in the public health literature by providing information subject to less retrospective recall bias than questionnaires.

In addition, ADAPT utilizes both state-of-the-art daily sleep diaries and actigraphy to capture subjective and objective information about sleep. The majority of studies examining sleep and weight gain are epidemiological and are limited by the use of global interview questions about sleep quality or sleep duration. Self-reported interview questions about sleep are limited in their ability to predict actual sleep [41], potentially due to retrospective recall bias. Moreover, global assessments do not adequately capture differences between the constructs of sleep duration and sleep fragmentation, each of which may be associated with different mechanisms towards poor health outcomes (e.g., see [94]).

The ADAPT design does not include a baseline assessment prior to job loss, since it is impossible to predict who will lose their job. Therefore, conclusions cannot be made about whether job loss caused disruptions in social rhythms or sleep. While it would be possible to compare unemployed and employed participants, this would dramatically limit the sample size for studying variations in stress exposure. Also, this modification would only answer limited questions about the effects of the event itself. Job loss is an entry event into a period of stress, but the patterns of stress disruption vary both across individuals and time. Much can be gained by understanding these dynamic fluctuations, since the effects of unemployment on weight gain already have substantial epidemiological support [4–6]. To address this concern, we explicitly examine the effects of reemployment and expect reemployment to occur for a substantial proportion of the sample, thereby providing an opportunity to study return to work as a point when re-stabilization should occur. Few studies have examined predictors of reemployment. In addition, the study includes psychological distress as a covariate at all time-points to assess whether depression and locus of control predict reemployment. We also include the gold-standard, time-intensive, Life Events and Difficulties Schedule to assess and control for contextual stressors (e.g., financial debt, loss of health insurance, use of government subsidy programs, housing insecurity) likely to occur and resolve as a function of job loss and later employment.

## Conclusions

The prevalence of obesity has been increasing over the past 30 years [95]. Many factors influence obesity beyond diet and physical activity. Two of the more prominent factors gaining scientific attention are sleep and stress. Poor sleep quality is highly prevalent in the U.S. [96], and the average sleep duration has steadily declined nationwide [97]. While studies have shown that stress is associated with disturbed sleep and that short sleep is associated with weight gain, no studies have examined how a disruption in daytime routine might impact sleep and weight gain. The proposed research is significant in its multivariate, prospective approach examining the inter-relationships between daily behavioral patterns, sleep, and weight gain in the aftermath of a stressful life event. The proposed study addresses three major U.S. social and public health concerns: unemployment, poor sleep, and obesity. The results from this study will have significant preventive implications in its (a) identification of unemployed individuals who are vulnerable to weight gain and (b) demonstration of sleep and social rhythms as mechanisms that are amenable to modification via behavioral intervention [98, 99].

## Abbreviations

AAFQ: Arizona activity frequency questionnaire
ACE: Adverse childhood experiences
ADAPT: Assessing daily activity patterns through occupational transitions
AHI: Apnea-hypopnea index
AZDES: Arizona Department of Economic Security
BAI: Beck anxiety inventory
BDI-II: Beck depression inventory
BIA: Bioelectrical impedance analysis
BMI: Body mass index
CCI: Charlson comorbidity index
DSD: Daily sleep diary
DSI: Duke sleep interview
LEDS: Life events and difficulties schedule
MAR: Missing at random
MEQ: Morningness eveningness questionnaire
METs: Metabolic equivalents
MINI: Mini International Neuropsychiatric Interview
PSQI: Pittsburgh sleep quality index
PSS: Perceived stress scale
RFPM: Remote food photography method
SE: Sleep efficiency
SOL: Sleep onset latency
SRD: Social rhythm disruption
SRM: Social rhythm metric
TIB: Time in bed
TST: Total sleep time
WASO: Wake time after sleep onset
